# World neglected tropical diseases day

**DOI:** 10.1371/journal.pntd.0007999

**Published:** 2020-01-29

**Authors:** Peter J. Hotez, Serap Aksoy, Paul J. Brindley, Shaden Kamhawi

**Affiliations:** 1 Departments of Pediatrics and Molecular Virology & Microbiology, Texas Children’s Hospital Center for Vaccine Development, National School of Tropical Medicine, Baylor College of Medicine, Houston, TX, United States of America; 2 Hagler Institute for Advanced Study at Texas A&M University, College Station, TX, United States of America; 3 Department of Biology, Baylor University, Waco, TX, United States of America; 4 James A Baker III Institute of Public Policy, Rice University, Houston, TX, United States of America; 5 Scowcroft Institute of International Affairs, Bush School of Government and Public Service, Texas A&M University, College Station, TX, United States of America; 6 Department of Epidemiology of Microbial Diseases, Yale School of Public Health, New Haven, CT, United States of America; 7 Department of Microbiology, Immunology, and Tropical Medicine, George Washington University School of Medicine, Washington, DC, United States of America; 8 Vector Molecular Biology Section, Laboratory of Malaria and Vector Research, National Institute of Allergy and Infectious Diseases, National Institutes of Health, Bethesda, MD, United States of America; Uniformed Services University, UNITED STATES

## Abstract

January 30, 2020 is the first-ever World Neglected Tropical Diseases Day (World NTD Day), a day when we celebrate the achievements made towards control of the world’s NTDs, yet recognize the daunting challenges we face in the control and elimination of these conditions.

Currently, the World Health Organization (WHO) identifies 20 major conditions as NTDs (Fig 1) [1], led in prevalence or incidence by the four major soil-transmitted helminth infections (ascariasis, hookworm infection, trichuriasis, and strongyloidiasis), followed by lymphatic filariasis, schistosomiasis, scabies, leishmaniasis, Chagas disease, and dengue. It is likely that all of the world’s population living below the World Bank poverty line of US$1.90 per day are infected with one or more of the WHO’s 20 NTDs, corresponding to at least 10% of the global population [2]. These NTDs also disproportionately affect indigenous populations living in extreme poverty [3].

**Fig 1:**
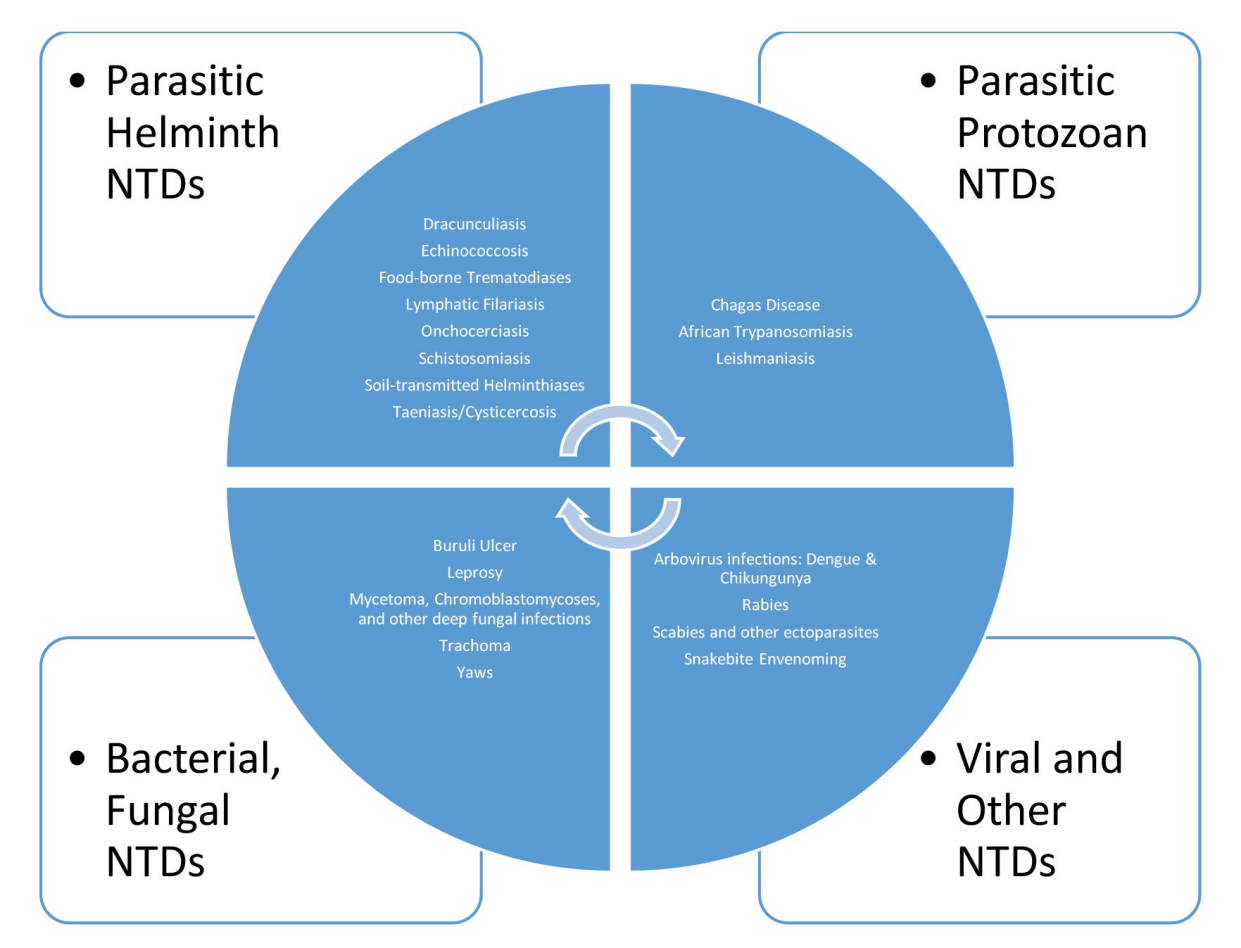
The WHO’s list of 20 Neglected Tropical Diseases (NTDs) [1].

With the exception of some of the arbovirus infections and rabies, the NTDs are generally described as chronic and debilitating illnesses with the ability to promote poverty because of their effects on productivity, child development, societal stigma, and the health of women. Based on those criteria, *PLOS Neglected Tropical Diseases* has expanded the WHO list to include a total of 40 NTDs [4].

## Scale-up interventions

Since 2005, the global health policymakers and community have branded the high-prevalence poverty-related neglected diseases as NTDs, recognizing both their co-endemicity and the opportunity this offers to target a group of them simultaneously through mass treatments–also known as preventive chemotherapy—with a package of low-cost interventions [5]. In 2007, the first issue of *PLOS Neglected Tropical Diseases* was launched, becoming the first open access journal committed to these diseases [6]. Later, in 2012, a London Declaration for NTDs redoubled global disease control efforts, securing long-term commitments from multinational pharmaceutical companies to donate essential medicines for NTDs, and ultimately establishing disease elimination targets [7].

A recent report from Uniting to Combat NTDs indicates that approximately $300 million in public and private funding is now committed annually to supporting packages of preventive chemotherapy, with an emphasis on seven NTDs–the soil-transmitted helminth infections, schistosomiasis, lymphatic filariasis, onchocerciasis, trachoma, yaws, and scabies [8]. The governments of the United States and United Kingdom currently provide the largest share of financial support for implementation, followed by the Bill & Melinda Gates Foundation and a private END Fund [8]. But other donors and partners are also beginning to contribute, with the recognition that mass treatment for NTDs represents an important “gateway” to achieving WHO goals for universal health coverage (UHC) [8]. Indeed, the WHO now estimates that more than one-half of the world’s poor receive preventive chemotherapy annually [9]. There is optimism that some preventive chemotherapy targets, such as trachoma, yaws, and lymphatic filariasis will be eliminated through this approach, while for the others it will be important to link preventive chemotherapy to water, sanitation, and hygiene (WASH) and accelerated economic development, in order to achieve sustainable disease burden reductions [10]. In addition, we are approaching the potential elimination of sleeping sickness in Africa [11] and visceral leishmaniasis on the Indian subcontinent [12].

Other NTDs have not benefited nearly as much from access to essential medicines or other interventions. For instance, it is estimated that only 1–2% of patients living with Chagas disease receive access to diagnosis and treatment [13], while millions of people lack access to leprosy diagnosis and treatment [14]. This is likely true for many other conditions on the WHO list of NTDs. Moreover, even for the NTDs targeted for preventive chemotherapy, the future of continued funding commitments from the US and UK governments is by no means assured, especially considering the current wave of populism sweeping these two nations and the re-emergence of isolationism [15]. Therefore, it will be essential to seek out new donors and partners. The finding that an unexpectedly large number of people living with NTDs include the marginalized poor living in the group of 20 (G20) economies [16], suggests that the G20 governments outside of the US and UK represent an important yet mostly untapped source of aid and support.

## A path forward

Each of the NTDs would currently benefit from the development of new and improved drugs, diagnostics and other technologies [17]. Even some of the diseases now targeted for control through preventive chemotherapy will require new technologies [17]. However, with the exception of the viral NTDs, especially dengue, none of the large multinational pharmaceutical companies are currently embarking on substantial initiatives to develop NTD control tools. A major reason for this observation is the simple fact that most of the NTDs only affect people who cannot afford new pharmaceuticals, and we do not yet have strong incentivizing mechanisms for large companies to invest in NTDs. While a priority review voucher (PRV) system has helped in a few instances, such as for the development of moxidectin for ochocerciasis [18], for the most part NTDs technologies are left to a fragmented ecosystem comprised of few product development parnterships (PDPs), such as the Drugs for Neglected Diseases Initiative (DNDi), PATH, and IVCC for vector control, academic laboratories, selected developing country vaccine manufacturers, and a BIO Ventures for Global Health (BVGH) accelerator. The current innovation system is not nearly adequate to develop the dozens of urgently needed control tools that will be required in the coming decades. This is tragic, because NTDs and NTD pathogens are now increasingly amenable to cutting edge technologies, including single RNA sequencing, OMICs, gene editing technologies, modeling, and systems biology [19]. Once again, most of the financing for NTD innovations relies on the US and UK governments, as well as the European Union, together with the Gates Foundation, but we urgently require the leaders of the other G20 nations to elevate their financial and intellectual contributions [16]. Recent public private partnerships between the Gates Foundation, local life sciences industries and the governments of Japan [16] and Korea [20] may offer some promise on this front.

We must recognize that an unstable world has created an uncertain future for the control and elimination of the world’s NTDs. The current funding stream for preventive chemotherapy is fragile and under threat, and the current innovation ecosystem for new technologies is inadequate. World NTD Day is a time to acknowledge our successes, but also to recognize that the world’s poor will likely continue to suffer from NTDs in the absence of continued and expanded commitments from global leaders, policymakers, and the donor community.
